# HYPofractionated Adjuvant RadioTherapy in 1 versus 2 weeks in high-risk patients with breast cancer (HYPART): a non-inferiority, open-label, phase III randomised trial

**DOI:** 10.1186/s13063-023-07851-7

**Published:** 2024-01-02

**Authors:** Budhi Singh Yadav, Divya Dahiya, P. Kannan, Shikha Goyal, Ishita Laroiya, Santhosh Irrinki, Ngangom Robert Singh, Reena Sharma

**Affiliations:** 1 Department of Radiotherapy & Oncology, Post Graduate Institute of Medical Education & Research, Chandigarh, India; 2 Department of General Surgery, Post Graduate Institute of Medical Education & Research, Chandigarh, India

**Keywords:** Breast cancer, Radiotherapy, Hypofractionation, Acute toxicity, Late toxicity, Recurrence, Quality of life, Survival

## Abstract

**Background:**

Breast cancer is the most common cancer in women. Radiotherapy is an important part of breast cancer treatment after surgery. Breast cancer radiotherapy is usually delivered in 3–5 weeks. This is a long duration for women with breast cancer to stay away from the family and work. We wanted to reduce this duration so that the wages loss and the logistics can be minimised for these patients. Hypofractionation, i.e. high dose per fraction, is delivered in a smaller number of days. In this study, we will compare a 1-week schedule of hypofractionated adjuvant whole breast/chest wall and/or regional nodal radiotherapy against 2 weeks for locoregional disease control, toxicities, quality of life (QoL), survival and second cancers after primary surgery in patients with breast cancer.

**Methods:**

Eligible patients with breast cancer after mastectomy or breast conserving surgery (BCS) will be treated with a radiotherapy dose of 26 Gy in 5 fractions over 1 week in the study arm and 34 Gy in 10 fractions over 2 weeks in the control arm. The primary endpoint of this noninferiority study will be locoregional tumour control. Secondary endpoints will be early and late radiation toxicities, quality of life, contralateral primary tumours, regional and distant metastases, survival and second cancers. A total of 1018 patients will be randomised (1:1) to receive 1 week or 2 weeks of radiotherapy. An event-driven analysis will be performed after at least 94 patients have documented locoregional recurrences. Acute radiation toxicity will be assessed and scaled according to the RTOG grading system. Late radiation toxicity will be assessed with the Radiation Therapy Oncology Group and the European Organisation for Research and Treatment of Cancer late radiation morbidity scale. Cosmetic assessment will be done using Harvard/NSABP/RTOG breast cosmesis grading scale at baseline and 3 and 5 years. QoL will be assessed with EORTC QLQ-30 and EORTC QLQ-BR 23 at baseline and 3 and 5 years.

**Discussion:**

Hypofractionation reduces treatment time to half while maintaining breast cosmesis and gives control rates equal to conventional fractionation. This is possible because breast tissue can tolerate high dose per fraction. In this study, we presume that 1-week radiotherapy will be non-inferior to 2 week radiotherapy, i.e. disease control will be similar with both the schedules without additional side effects, and QoL of these patients will be maintained. If we are able to achieve these outcomes, then patients will be able to complete their radiotherapy in less duration. There is not much data on regional nodal irradiation with hypofraction in breast cancer. We have used hypofraction for regional nodal irradiation in the past and not encountered any safety issue. If we are able to prove that late-term effects are comparable in the two schedules, it will make the radiation oncologist confident about hypofractionation in breast cancer. As breast cancer is a leading cancer in females and radiation therapy is an integral part of its local management, hypofractionation will help radiation centres worldwide to meet the growing need for radiation treatment in breast cancer, particularly in developing countries where resources are limited. It will also reduce the financial burden on the patient and family. Since we will treat these patients with both simple and complex radiotherapy techniques, it will also be possible for the low-income countries to follow this trial without needing a high-end or expensive radiotherapy equipment as the planning and treatment process will be very simple.

**Trial registration:**

The trial is registered with ClinicalTrials.gov ID NCT04472845 and CTRI with REF/2020/09/037050.

**Supplementary Information:**

The online version contains supplementary material available at 10.1186/s13063-023-07851-7.

## Background

In the past, standard radiotherapy for breast cancer was 50 Gy of dose delivered in 25 fractions over 5 weeks. This is called conventional fractionation; here, dose per fraction is 2 Gy. Sometimes, in young patients and those with high-risk disease, this is followed by a boost dose of 10–20 Gy in 5–10 fractions delivered over 5–10 days, which increased this treatment duration to 6–7 weeks, which is a long time. Hypofractionation is high dose per fraction delivered in a smaller number of days. We at PGIMER, Chandigarh, India, have been practicing hypofractionated radiotherapy in breast cancer patients for the last 5 decades. Our standard doses have been 35 Gy/15#/3wks to the chest wall after mastectomy and 40 Gy/16#/3wks after breast conserving surgery (BCS). We have published our results with moderate hypofractionated radiotherapy (35–40 Gy delivered in 15 fractions over 3 weeks) in patients with breast cancer and observed comparable favourable clinical outcomes [1–6]. Hypofractionation is also a routine practice in the UK and in a few centres in Canada [7, 8]. A study from Beijing also reported similar outcomes with conventional and hypofractionated radiotherapy [9]. The current standard for hypofractionation in breast cancer is 40–42.5 Gy delivered in 15–16 fractions over 3 weeks followed by a boost of 10–16 Gy in 5–8 fractions delivered over 1–2 weeks [6]. This is also called moderate hypofraction. Moderate hypofractionation may not be the upper limit of hypofractionation in breast cancer because breast tissue has low α/β ratio of 3.5 [7]. The ratio α/β is the dose at which the linear and quadratic component of cell killing are equal. α damage leads to single strand break in DNA which might be repairable, whereas β damage leads to double strand DNA damage which is not repairable. β damage occurs at high dose per fraction. This is a concept of radiobiology where α/β is assumed to be 3 Gy for late-responding tissue and 10 Gy for early responding tissue. With α/β of 3.5 for the breast, it behaves like a late responding tissue, which means high dose per fraction (hypofraction) is more suitable to treat breast cancer. So, accelerated hypofractionation (34 Gy delivered in 10 fractions over 2 weeks) is also a possibility in breast cancer [10–13]. With this background, we think that it is possible to reduce this treatment duration further. A recent study has also shown that 1-week treatment is similar to 3-week treatment in patients with early breast cancer [14]. We have completed a trial where we compared a 3-week schedule to 2-week and observed similar acute toxicity but cosmetic outcome was better with 2-week schedule [15]. Therefore, we chose the 2 weeks schedule as a comparator arm.

In this study, we want to test a 1-week schedule of hypofractionated adjuvant whole breast/chest wall and/or regional nodal radiotherapy against 2 weeks for locoregional control, acute and late toxicities, QoL, survival and second cancers after primary surgery in patients with breast cancer.

## Methods/design

We used the SPIRIT reporting guidelines (Supplementary file) [16]. This study will be a randomised, non-inferiority, open-label, phase 3 trial, with patients to be recruited from a single institute. Other institutes will be invited if they are willing to participate. Patients to be included in this study will be pre-operatively staged according to American Joint Committee on Cancer (AJCC) 8th edition, International Union against cancer (which uses TNM staging), as stage III breast carcinoma. For patients who receive adjuvant chemotherapy, pathological stage will be reflected, and for those with neoadjuvant chemotherapy, the stage which is higher (clinical/pathological) will be reflected. A total of 1018 patients with histologically proven post lumpectomy/mastectomy cases of carcinoma breast suitable for radiotherapy will be enrolled in this study. Patients will be evaluated at the Department of Radiation Oncology Post Graduate Institute of Medical Education & Research, Chandigarh, India. A thorough clinical examination followed by routine investigations which will include hemogram, liver function tests, kidney function tests, mammography, chest X-ray, ultrasound of abdomen/CECT chest, abdomen and pelvis and whole-body bone scan. At first visit, the patient will be screened, and if she/he agrees, the written informed consent will be taken from all the patients by BSY, residents/clinical associates. If the patient needs more time to think over the study, then the consent will be taken in the subsequent visit. Patients must meet all of the following inclusion criteria and none of the exclusion criteria:

Inclusion criteria

1. Age ≥ 18–75 years
2. Female or male
3. Invasive carcinoma of the breast
4. Breast conserving surgery (BCS) with axillary clearance or total mastectomy with axillary clearance (TMAC) (reconstruction allowed but not with implant; tissue expanders with distant metal ports are allowed)
5. Concurrent trastuzumab and hormone therapy is allowed
6. Axillary staging and/or dissection
7. Complete microscopic excision of primary tumour
8. pT3-4, pN2-3, M0 disease
9. Clinical stage III disease or pathological node positive if they have received neo-adjuvant chemotherapy
10. Written informed consent
11. Able to comply with follow-up

Exclusion criteria

1. Supraclavicular node or internal mammary node or distant metastasis
2. Past history of malignancy except (i) basal cell skin cancer and CIN cervix uteri or (ii) non-breast malignancy allowed if treated with curative intent and at least 5 years disease free
3. Contralateral breast cancer, including DCIS, irrespective of date of diagnosis
4. Breast reconstruction using implants
5. Pregnancy (a sexually active fertile female needs to agree to use contraceptives)
6. Concurrent cytotoxic chemotherapy (sequential neoadjuvant or adjuvant cytotoxic therapy allowed)

### Randomisation and masking

Eligible patients will be randomly assigned (1:1) to receive either 1 week or 2 weeks of radiotherapy without stratification by simple randomisation according to a prescribed computer-generated central randomisation schedule. Radiation oncologists in the study team will enrol participants, and research staff members who will be involved in follow-up data collection will assign participants to interventions. Allocation concealment will be ensured by not releasing the randomisation code until the patient has been recruited into the trial by using concealed opaque envelops. Treatment allocation will not be masked but outcome assessors will be blinded to reduce bias. Unblinding will be permitted in case of serious adverse events. Patients who want to withdraw or change their treatment schedule will be treated with 34 Gy in 10 fractions over 2 weeks or 40 Gy in 15 fractions over 3 weeks. All data entered will be checked by BSY, DD and PK.

### Radiotherapy planning

For 2-dimensional (2D) planning, patients will be in supine position on a breast board with ipsilateral arm abducted to 90°. Patients will be planned using 2-D fluoroscopic conventional simulator with two tangential fields to breast/chest wall and a single incident field to supraclavicular fossa. Field marking for breast/chest wall will include midline medially, midaxillary fold laterally, 2nd intercostal space cranially and 1 cm below opposite inframammary fold caudally. For supraclavicular field, caudal border will be cranial border of breast/chest wall field, cranially thyroid notch, medially along medial border of sternocleidomastoid muscle and laterally insertion of deltoid. Central lung distance (CLD) will be recorded for all patients. Wedge of 30° will be used in patients with BCS.

For conformal techniques, a CT-based three-dimensional planning will be used to reconstruct radiotherapy target volume for treating breast/chest and locoregional lymph nodes. The patients will be positioned supine on a breast board with arm abducted above the head on arm rest in planning CT in radiotherapy department. CT axial cuts will be taken from the level of larynx to upper abdomen, including both the lungs with a scan thickness and index of 3 mm. CT images will be transferred to the treatment planning system (TPS). The chest wall or whole breast, heart, bilateral lungs and opposite breast will be contoured using Radiotherapy Oncology Group (RTOG) contouring guidelines. For left anterior descending (LAD) coronary artery contouring, the left coronary artery will be identified, and its course was followed. LAD will be contoured from the place where the it branches away from the main left coronary artery and then runs towards the front and down towards the heart apex. It will be divided in to proximal and distal parts. Distal LAD will be the part which lies very near to the chest wall towards the apex of the heart. No additional margin will be given to LAD. The treatment parameters, patients and OARs outlines will be exported to computerized TPS, and plans will be made using standard tangent fields.

Radiotherapy dose to chest wall, axilla level III and supraclavicular fossa will be of 26 Gy in 5 fractions over 1 week in the study arm and 34 Gy in 10 fractions over 2 weeks in the control arm. BCS patients will receive a sequential boost of 8 Gy/2#/2days or simultaneous integrated boost (SIB) to a total dose of 32 Gy in the study arm and 40 Gy in the control arm. Treatment will be done on a linear accelerator with 6 or 10 MV photon energy. Boost can be with photons or electrons. One-centimetre bolus will be used in all postmastectomy patients, daily in study arm and in 50% of treatment in the control arm. Dose will be prescribed at mid-separation in 2D technique and the dose covering 95% of the planning target volume with three-dimensional conformal (3D-CRT) or intensity-modulated radiotherapy (IMRT).

Supraclavicular fossa (SCF) and axilla level III will be treated in patients with T3-4 disease with lymphovascular invasion, grade 3 or N2 disease and T3-4 disease treated with neoadjuvant chemotherapy after adequate axillary dissection. Level I and II axilla will be irradiated in patients with inadequate axillary dissection (< 10 lymph nodes) and if there is extracapsular extension. The prescribed dose to the area will be defined as the dose to the point at 3 cm beneath the skin in two-dimensional radiotherapy technique or the dose covering 95% of the planning target volume with 3D-CRT or IMRT.

Internal mammary node (IMNs) radiation will be done in T3-4 central and inner quadrant lesions, patients with N2 disease and in patients having IMN on imaging. IMNs will be irradiated with a separate 12 × 5 cm single field. The first three intercostal spaces will be included in the IMN target volume. The medial border of the IMN field will be midline; lateral border 4 cm lateral to the midline; the superior border will abut the inferior border of the supraclavicular field; and the inferior border will be above the xiphoid. Dose will be 34 Gy/10#/2weeks in the control arm and 26 Gy/5#/1 week in the study arm. Dose will be prescribed at 3 cm depth. IMNs can be treated with electrons or photons with 2D/3D/IMRT techniques. Imaging will be done for set-up verification on alternate days in the study arm and once weekly in the control arm.

Hormonal therapy may be given to oestrogen receptor-positive or progesterone receptor-positive tumours and anti-HER2-targeted therapy (e.g. trastuzumab) for those with HER2-positive disease depending on the affordability.

### Assessment

**Fig. 1:**
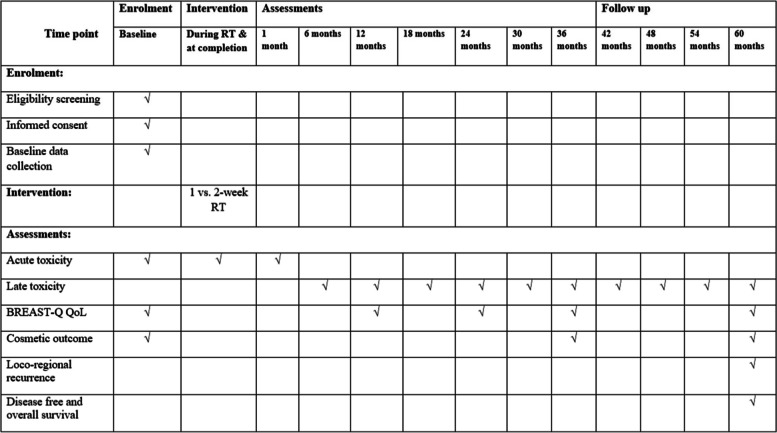
Patient assessment

Patients will be assessed for medical history, a physical examination and a blood test before radiotherapy, once per week during radiotherapy and 2 weeks after radiotherapy (Fig. 1). Patients will be followed up every 3 months for 2 years after radiotherapy, then every 6 months from 3 to 5 years, and yearly thereafter. Skin, subcutaneous toxicity and cosmetic assessment will be done before treatment and then in regular follow-up of the study. Acute radiation toxicity will be assessed and scaled according to the RTOG grading system (Table 1). Acute toxicity will be accessed weekly during radiotherapy, at the time of completion and at 1 month after completion of radiotherapy. Late radiation toxicity will be assessed with the Radiation Therapy Oncology Group and the European Organization for Research and Treatment of Cancer (EORTC) late radiation morbidity scale at 6 months, 1 year, 2 years, 3 years, and 5 years. Cosmetic assessment will be done using Harvard/NSABP/RTOG breast cosmesis grading scale at baseline and 3 and 5 years. QoL will be assessed with EORTC QLQ-30 and EORTC QLQ-BR 23 at baseline and 3 and 5 years. Any adverse event will be communicated to the IEC. Patients will be reminded for follow-up by scheduling appointments and monitoring. Missed patients will be contacted by telephone, mails and written letters.

**Table 1:** RTOG acute and late toxicity grading scale

Organ tissue	0	1	2	3	4	5
Skin (acute)	No change over baseline	Follicular, faint or dull erythema/epilation/dry desquamation/decreased sweating	Tender or bright erythema, patchy moist desquamation/moderate oedema	Confluent, moist desquamation other than skin folds, pitting oedema	Ulceration, haemorrhage, necrosis	NA
Skin (late)	None	Slight atrophy; pigmentation change; some hair loss	Patch atrophy; moderate telangiectasia; total hair loss	Market atrophy; gross telangiectasia	Ulceration	Death directly related to radiation late effects
Subcutaneous tissue	None	Slight induration (fibrosis) and loss or subcutaneous fat	Moderate fibrosis but asymptomatic; slight field contracture; < 10% linear reduction	Severe induration and loss of subcutaneous tissue; field contracture > 10% linear measurement	Necrosis	
Lung	None	Asymptomatic or mild symptoms (dry cough) Slight radiographic appearances	Moderate symptomatic fibrosis or pneumonitis (severe cough) Low-grade fever; patchy radiographic appearances	Severe symptomatic fibrosis or pneumonitis Dense radiographic changes	Severe respiratory insufficiency/continuous 02/assisted ventilation	
Chest or rib pain	None	Asymptomatic; no growth retardation; reduced bone density	Moderate pain or tenderness; growth retardation; irregular bone sclerosis	Severe pain or tenderness; complete arrest of bone growth; dense bone sclerosis	Necrosis/spontaneous fracture	
Shoulder morbidity	None	Mild joint stiffness; slight limitation of movement	Moderate stiffness; intermittent or moderate joint pain; moderate limitation of movement	Severe joint stiffness; pain with severe limitation of movement	Necrosis/complete fixation	
Cardiac	None	Asymptomatic or mild symptoms; transient T wave inversion & ST changes; sinus tachy> 110 (at rest)	Moderate angina on effort; mild pericarditis; normal heart size; persistent abnormal T wave and ST changes; low ORS	Severe angina; pericardial effusion; constrictive pericarditis; moderate heart failure; cardiac enlargement; EKG abnormalities	Tamponade/severe heart failure; severe constrictive pericarditis	

### Data management

Case record forms (CRFs) will be filled by the clinical associates, and data will also be entered on an excel sheet by logging in. It will be supervised by the investigator. CRFs will be tracked for missing pages and illegible data manually to assure that the data are not lost. Quality control of the data will be done at regular interval. Medical coding of the data will be done. Data will be stored in the Radiotherapy & Oncology Department in the clinical trial room, in a designated computer, with access to the clinical associate and the investigator.

### Outcomes

The principal end point of the study is locoregional recurrence, defined as disease recurrence in the ipsilateral chest wall or regional lymph nodes from the time of randomisation until the end of follow-up.

Secondary end points include disease-free survival, overall survival, acute and late radiation toxicities cosmetic score analysis, QoL and second cancers. Overall survival events will be defined as death from any cause from the time of randomisation until the end of follow-up. Disease-free survival events will be defined as locoregional recurrence, distant metastasis or death from any cause from the time of randomisation until the end of follow-up.

### Statistical considerations

The primary hypothesis of the study is that locoregional recurrence with 1 week is non-inferior to that with 2 weeks of radiotherapy. The primary endpoint will be locoregional recurrence (LRR). Based on historical data (Wang et al.), the 5-year LRR rate with 3 weeks of RT is expected to be approximately 8%. One week of RT would be considered non-inferior to 2 weeks of RT if the 5-year LRR rate does not exceed 13%. If LRR follows exponential distributions within each treatment group, this non-inferiority margin corresponds to a hazard ratio (HR) of 1.67.

Sample size calculations were performed using module STE0S-3 of nQuery version 8.5.2.0. A total of 94 LRR events will be required to provide at least 80% power using a one-sided test at the 5% level of significance. This would be a time-to-event analysis, and patients will be randomised 1:1 to observe the required (94) LRR events after 3 year or accrual and 5 years of follow-up. Under assumed failure rates and guarding against 10% ineligibility or loss to follow-up, the target accrual will be 1018 patients with an accrual period of 3 years. If the two schedules are equivalent, 5 years of follow-up after the last patient has been randomised will be required to observe 94 LRR events, resulting in total study duration of 8 years. For achieving adequate participant enrolment to reach the target sample size, patients will be recruited continuously until the desired sample size is achieved, and all investigators would be encouraged for patient referral.

For the primary efficacy end point, we will use the cumulative incidence method to estimate the actual incidence of locoregional recurrence at different time points, with death without locoregional recurrence as a competing risk. We will use the log-rank test and Cox regression model for statistical inference based on cause-specific hazards. We will calculate overall survival and disease-free survival using the Kaplan-Meier method, and we will analyse these endpoints using the log rank test and Cox regression model. Acute and late toxicities will be summarised as frequency and severity on the basis of their association with protocol treatment; *χ*^2^ tests will be used to compare the differences. The primary endpoint and all efficacy endpoints will be analysed in a modified intention-to-treat population (i.e. including all eligible patients who will undergo randomisation but exclude those who are considered ineligible). On the basis of the study design, the primary endpoint locoregional recurrence will be analysed at a one-sided significance level of 0.05 and reported with a two-sided 90% CI. All other statistical tests will be two sided, and *p* values of < 0.05 will be taken as significant. All tests will be performed using SPSS (Statistical Package for the Social Sciences) v.23.0.

No interim analysis is planned. Any adverse outcome or toxicity from the radiation schedules will be reported to the IEC. Final decision to terminate the trial lies with the IEC. Patient confidentiality will be maintained, and re-identifiable-identifiers will be removed and replaced by a code.

## Discussion

Hypofractionation reduces treatment time to half while maintaining cosmesis and gives control rates equal to conventional fractionation. As breast cancer is a leading cancer in females and radiation therapy is an important part of its local management, hypofractionation will help radiation centres worldwide to meet the growing need for radiation treatment in breast cancer, particularly in developing countries where resources are limited and breast cancer incidence is increasing. It also reduces the financial burden on the patient and family.

Getting funding for the clinical trials is a huge task. Two funding agencies have not approved the funds. We have applied to a third funding agency which have agree for the same. COVID-19 posed a challenge and affected patient care all over the world [17, 18]. Because of COVID-19, initial recruitment was slower in the trial, but now recruitment is going smoothly. We are also planning to involve other institutes to make it a multicentre study. So far, three institutes have agreed to participate in the study; hopefully, we will be able to complete the recruitment earlier than the projected.

Sometimes, there are technical challenges such as radiation machine break-up and to maintain the quality assurance. Timely intervention in such a situation such as to treat the patients in other machine with the same technique and regular quality assurance will be ensured. Breast cancer patients survive longer. Radiation toxicities concern with hypofractionation, especially late-term effects [19, 20]. Such patients will be kept on stringent surveillance. But we have not encountered excess adverse toxicities with moderate and accelerated hypofractionation in our patients with breast cancer [4, 12, 14]. We have used hypofraction in very young [21], young [22] and elderly patients with breast cancer [23] and even male patients [24] and observed late toxicities and survival outcomes similar to those reported with conventional fractionation. There is not much data on regional nodal irradiation with hypofraction in breast cancer. We have used hypofraction for regional nodal irradiation in the past and not encountered any safety issue [25]. So, with this experience, we have some confidence that these schedules may be safe and efficacious.

### Trial status

Protocol version 2 dated 10 March 2021. The date of recruitment is 1 April 2021, and the approximate date when the recruitment will be completed is 31 March 2025.

### Supplementary Information

**Additional file 1.**

**Additional file 2.**

**Additional file 3.**

**Additional file 4.**

**Additional file 5.**

## Data Availability

Data will be provided or shared on a suitable request forwarded to drbudhi@gmail.com.
