# Supplementary material for: HYPofractionated Adjuvant RadioTherapy in 1 versus 2 weeks in high-risk patients with breast cancer (HYPART): a non-inferiority, open-label, phase III randomised trial
Source: Trials. 2024 Jan 2;25:21. doi: 10.1186/s13063-023-07851-7 (PMC10763219; doi:10.1186/s13063-023-07851-7)
Supplement: Supplementary file 1 — Additional file 1. [file 13063_2023_7851_MOESM1_ESM.doc]

***TRIAL SCHEMA***

# HYPofractionated Adjuvant RadioTherapy in 1 versus 2 weeks in high-risk patients with breast cancer (HYPART):A non-inferiority, open-label, phase III randomized trial.

**SCREENING & ACCRUAL**

- Screen at in the OPD 4003C on Monday, Wednesday & Friday
- Consenting to be done before patient starts radiotherapy
- Confirm eligibility: All patients >18 years, female/male, pT3-4 N2-3 M0 disease
- Exclusion: pregnancy, previous H/O malignancy, bilateral breast cancer, unfit for treatment
- Eligible: NO Enter details in screening register with reasons

YES NOT WILLING

WILLING MORE THAN ONE COMPETITIVE TRIAL(QoL)

NO STUDY OR ONLY ONE STUDY SERVE ICF & CONSENTING

- Literate patient can sign the ICF alone, if patient illiterate, then literate attendant to sign along with patient’s thumb impression. Both illiterate then impartial witness to read out and also sign ICF along with thumb impression of both the patient and attendant.
- Take signatures/thumb impression on TWO pages if willing for giving blood and tissue archival.
- Keep the signed ICFs securely in the trial box folder

**RANDOMIZATION**

- Randomization takes place in CRS, Main building Ground floor
- Fill the **randomization CRF** before going to CRS and ensure it is completely filled.
- If cerb2 status is 2+ on IHC, then FISH needs to be done BEFORE RANDOMIZATION (from trial account)

**FOLLOW UP**

- Weekly during RT, at 1 month and 3 months of completion of radiotherapy
- Patients severe radiation reaction will be followed 2 weekly after reporting of reaction.
- Follow up (FU) 3 monthly till 1 year, 4 monthly till 2 year and 6 monthly till 5 years and yearly thereafter.
- Medical history, examination and late morbidity (can refer to morbidity CRF) at each FU.
- **QOL forms:** 0, 6, 12, 24, 36 and 60 months.
- **Mammogram of contra-lateral breast** at 18, 36 and 60 months.
- **Follow up CRF** to be filled for all patients at each FU
- **Death or recurrence CRF** to be filled in case of event.
