# Supplementary figures and images for: HYPofractionated Adjuvant RadioTherapy in 1 versus 2 weeks in high-risk patients with breast cancer (HYPART): a non-inferiority, open-label, phase III randomised trial
Source: Trials. 2024 Jan 2;25:21. doi: 10.1186/s13063-023-07851-7 (PMC10763219; doi:10.1186/s13063-023-07851-7)

Gantt chart

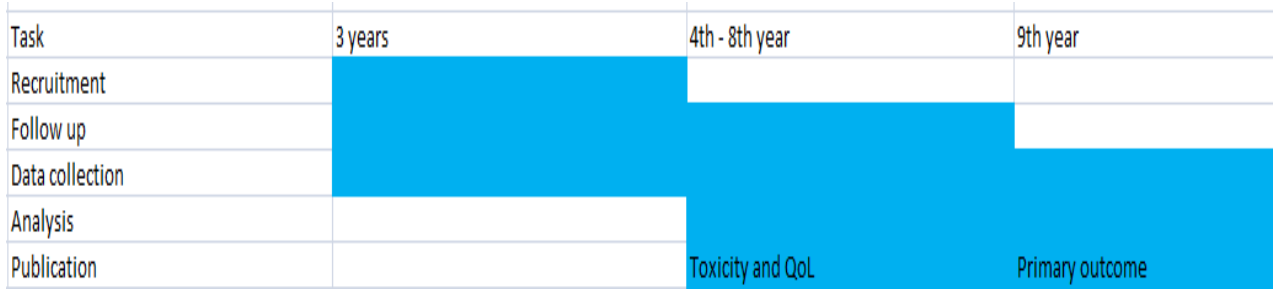

Supplement: Supplementary file 2 — Additional file 2. [file 13063_2023_7851_MOESM2_ESM.pdf]
