# Supplementary material for: HYPofractionated Adjuvant RadioTherapy in 1 versus 2 weeks in high-risk patients with breast cancer (HYPART): a non-inferiority, open-label, phase III randomised trial
Source: Trials. 2024 Jan 2;25:21. doi: 10.1186/s13063-023-07851-7 (PMC10763219; doi:10.1186/s13063-023-07851-7)
Supplement: Supplementary file 3 — Additional file 3. [file 13063_2023_7851_MOESM3_ESM.docx]

|  | **Baseline**  **(Post-op or post chemo)** | **During RT & at conclusion** | **6 months** | **12 months** | **18 months** | **24 months** | **30 months** | **36 months** | **42 months** | **48 months** | **54 months** | **60 months** |
| --- | --- | --- | --- | --- | --- | --- | --- | --- | --- | --- | --- | --- |
| **Informed consent for trail and archival of biological material** | √ |  |  |  |  |  |  |  |  |  |  |  |
| **Blood for future biological studies** | √ |  |  |  |  |  |  |  |  |  |  |  |
| **Medical history and examination** | √ | √ | √ | √ | √ | √ | √ | √ | √ | √ | √ | √ |
| **RTOG acute radiation morbidity scoring** | √ | √ |  |  |  |  |  |  |  |  |  |  |
| **RTOG late radiation morbidity scoring** |  |  | √ | √ | √ | √ | √ | √ | √ | √ | √ | √ |
| **BREAST-Q QOL forms** | √ |  | √ | √ |  | √ |  | √ |  |  |  | √ |
| **Additional morbidity data** | √ | √ | √ | √ | √ | √ | √ | √ | √ | √ | √ |  |
| **Radiologic assessment**  **of contra-lateral breast** | √ |  |  |  | √ |  |  | √ |  |  | √ |  |

**EVALUATION SHEDULE FOR HYPART TRIAL**
