# Supplementary material for: HYPofractionated Adjuvant RadioTherapy in 1 versus 2 weeks in high-risk patients with breast cancer (HYPART): a non-inferiority, open-label, phase III randomised trial
Source: Trials. 2024 Jan 2;25:21. doi: 10.1186/s13063-023-07851-7 (PMC10763219; doi:10.1186/s13063-023-07851-7)
Supplement: Supplementary file 4 — Additional file 4. [file 13063_2023_7851_MOESM4_ESM.doc]

**HISTORY, EXAMINATION AND DIAGNOSIS**

1. Age years

2. Menopausal status Pre Peri (Last period within 1 year) Post

3. Last menstrual period Day Month Year

4. Co-morbidities: Yes No. If Yes please specify _____________________

5. Literate Yes No. Qualification ___________________________

6. Mother tongue _____________________

7. Proficiency English Hindi Marathi

8. Laterality Right Breast Left Breast

9. Clinical Tumor Size: . cm (Maximum diameter of largest invasive component)

10. Prior Intervention: FNAC Core / Gun Bx

Date of prior intervention: Day Month Year

11. Histological Type: IDC DCIS ILC MRB Score:

12. Loco-regional examination:

**
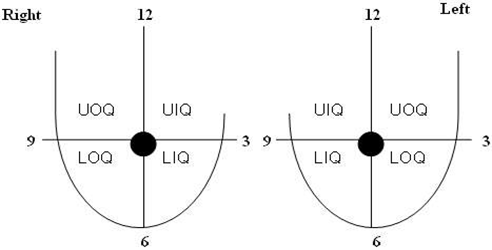
**

13. Clinical stage: cT cN M

**INVESTIGATIONS:**

1. Hematology and Biochemistry Day Month Year

1. Absolute neutrophil counts (ANC )≥ 1.5x 10e9/L (1500 /mm3) . . 10e9/L
2. Platelet count ≥ 100 x 10 e9/L ( 100,000 / mm3) . 10e9/L
3. Hemoglobin ≥ 9.0g/dL ( 90 g/L) . g/DL
4. Serum creatinine ≤ 1.5 x upper limit of normal (UNL) . . mg/dl
5. Total bilirubin ≤ 1.5 x UNL (<3 UNL if Gilbert’s disease) . . mg/dl
6. Aspartate aminotransferase (AST) SGOT ≤ 2.5 x UNL . . U/L
7. Alanine aminotransferase (ALT) SGPT ≤ 2.5 x UNL . . U/L

2. Cardiac function

MUGA ECHO Date Day Month Year

LVEF %

3. Metastatic work up

| Investigations | Date | Findings |
| --- | --- | --- |
| Mammography |  |  |
| Chest Imaging  (CXR/CT scan) |  |  |
| Abdominal imaging (USG/CTscan) |  |  |
| Bone Imaging  (MDP Bone scan /  F18 PET/FDG PET) |  |  |

**PHYSICAL EXAMINATION**

1. Blood Pressure (mm Hg): Systolic _________ Diastolic ____________
2. Height (cms): __________ Weight (Kgs): ___________
3. Pulse (minutes): ________ Regular / Irregular
4. ECOG Performance Status [Put X in the appropriate box]
   1. O - Fully active, able to carry out all pre-disease performance without

restriction.

b. 1 – Restricted in physically strenuous activity, but ambulatory and able to

carry out work of a light or sedentary nature.

c. 2 – Ambulatory and capable of all self-care, but unable to carry out any

work activities; up and about more than 50% of waking hours.

d. 3 – Capable of only limited self-care, confined to bed or chair; requires

assistance more than 50% of waking hours.

e. 4 – Completely disable, cannot carry on any self-care; totally confined to

bed or chair.

f. 5- Dead.

**Investigator/Designee**  Day Month Year

**Signature**
